# Sociodemographic Characteristics and Dietary Choices as Determinants of Climate Change Understanding and Concern in Saudi Arabia

**DOI:** 10.3390/ijerph182010605

**Published:** 2021-10-10

**Authors:** Ghada Talat Alhothali, Noha M. Almoraie, Israa M. Shatwan, Najlaa M. Aljefree

**Affiliations:** 1 Department of Marketing, College of Business, University of Jeddah, Jeddah 3795, Saudi Arabia; 2 Food and Nutrition Department, Faculty of Human Sciences and Design, King Abdulaziz University, Jeddah 3270, Saudi Arabia; nalmorie@kau.edu.sa (N.M.A.); eshatwan@kau.edu.sa (I.M.S.); naljefree@kau.edu.sa (N.M.A.)

**Keywords:** climate change, climate-friendly food, environmental concern, dietary choices, socio-demographics, greenhouse gas emissions, awareness, public health, Saudi Arabia

## Abstract

Climate change poses a global threat to public health. This study investigated the understanding of, and concern over, climate change in Saudi Arabia and examined the associations with sociodemographic characteristics and dietary choices. This cross-sectional study consisted of 280 participants recruited via an online survey. Of the study participants, 45% demonstrated a sufficient understanding of climate change, and 56% were highly concerned about climate change. Male sex, medium-high monthly income, high education, and governmental employees were determinants of sufficient understanding of and great concern over climate change. Participants who exhibited a high understanding of climate change score demonstrated significantly higher consumption of vegetables (3.47 ± 0.98) and vegetable oils (3.26 ± 1.07) than participants with a low understanding score (3.31 ± 0.96 and 3.00 ± 1.01, respectively) (*p* ≤ 0.01). Additionally, participants with higher concern of climate change scores exhibited lower consumption of red meat (*p* = 0.0001), poultry (*p* = 0.003), margarine (*p* = 0.02), and soy products (*p* = 0.04). The study revealed a poor understanding of, but great concern over, climate change. The intake of non-climate-friendly food was typically higher than that of climate-friendly food. These findings are critical for developing strategies to enhance awareness of climate change and encourage people to consume climate-friendly food to mitigate climate change and improve public health.

## 1. Introduction

Climate change is a global threat that affects the planet and an individual’s quality of life. It is considered one of the pivotal problems of the 21st century [1]. Scholars and policymakers worldwide are working to lessen the impact of climate change [1,2,3]. In the Intergovernmental Panel on Climate Change (IPCC) report, the scientific community revealed that greenhouse gas emissions (GHGEs) have escalated over the past few decades in spite of an increasing number of efforts to solve this issue [4]. The numerous harmful effects of climate change include the melting of ice sheets, increasing sea levels, and dangerous weather events as a result of severe heat waves. Similarly, land degradation, water scarcity and pollution, pose threats to future generations in terms of hunger and food security; thus, urgent attention should be demanded [1,4].

The devastating effects of human beings’ patterns of living on the environment cannot be ignored. One significant example is the substantial impact of food choices and consumption patterns on climate change [2,5,6]. Prior studies have shown that dietary choices have a substantial effect on the environment [7] and are considered a key strategy to mitigate climate change [4,8]. In particular, food consumption accounts for a significant amount of GHGEs [3]. Prior studies have also found that food production processes contribute to GHGEs by 20–30% [5,9]. Furthermore, previous studies have demonstrated that minimizing meat consumption by substituting organic and vegetarian food has decreased the GHGEs in Sweden and China [9,10]. This is because meat generates higher emissions per unit of energy due to the energy lost at each trophic level compared with plant-based foods [7]. Hence, the relationship between food intake and climate change are evidenced in prior studies, e.g., [1,2,4,5,8,9,10,11,12].

Recently, research evidenced that food-related carbon savings come primarily from dietary changes, particularly the adoption of a vegan diet [6]. According to a study that examined individual food choices and measured their potential impact on reducing GHGEs in developed countries, eating a plant-based diet can reduce annual personal emissions by 0.8 t CO_2_e/yr [6]. The environmental impacts of dietary choices can be minimized by decreasing animal product consumption, energy intake, and food waste [4].

The path toward a sustainable society can be pursued through governmental efforts and innovations and the sustainable actions of human beings. Hence, a cultural change is recommended for sustainable development [13].

Concerns over climate change have led to climate-friendly food consumption, involving the entire food consumption supply chain; from selecting, purchasing, cooking, and sorting food to disposing and recycling food waste. Climate-friendly food consumption refers to an individual’s voluntary actions limiting dairy and meat product intake to help protect the environment [6]. The extant literature showed that adopting climate-friendly food is among the mitigating strategies for climate change impact [12,13,14]. Prior studies have also demonstrated that individual awareness of food choices and their subsequent impact on the environment is growing [15,16]. A recent study by Jalil et al. [17] investigated the effects of an awareness-raising intervention on meat consumption through a randomized controlled trial. The study found that interventions that advise individuals and support voluntary change can substantially decrease meat consumption [17].

In western countries such as Scotland, Australia, and Finland, the majority of previous research focused on people’s awareness of food choices and their environmental impact [2,11,12]. Developing countries, on the other hand, have only conducted a small number of studies. Furthermore, there is still a lack of evidence linking climate change awareness and concern with subsequent mitigation behaviors in developing countries such as Saudi Arabia [18]. This study closes the knowledge gap.

This problem is worsening in Saudi Arabia, where dietary products are more prevalent in Saudi individuals’ daily food choices. Prior research investigated female food choices in the Eastern region of Saudi Arabia using qualitative methods and found that females are unaware of the impact of their food choices on environmental sustainability [19]. Accordingly, in a developing country such as Saudi Arabia, few studies have examined the understanding of, and concern about, climate change and its association with dietary choices [18]. Hence, this research investigated an individual’s understanding of, and concerns over, climate change in Saudi Arabia. The study also examines the associations between sociodemographic characteristics and dietary choices with the understanding of, and concern over, climate change within the Saudi population.

## 2. Materials and Methods

The current research is underpinned by the positivist paradigm, a school of thought which believes that only knowledge gained through observation and measurement can be trusted [20]. Studies based on positivism limit the researcher’s responsibilities to data collection and objective interpretation. This paradigm assists positivist researchers in better understanding phenomena through empirical methods such as sampling, measurement, and questionnaires. Positivists are primarily associated with quantitative research, since it involves gathering and converting data into numerical form to perform statistical calculations [20]. Hence, the use of a questionnaire as a primary tool of data collection is justifiable.

### 2.1. Study Population

This cross-sectional study included 280 participants between the ages of 18 and 60 years. Study participants were recruited from March to June 2021 in Jeddah City, Saudi Arabia. Recent statistical reports on Saudi Arabia based on Unified National data indicated that the total population in Jeddah in 2019 was 2,867,446 [21]. Therefore, the sample size required to obtain sufficient statistical power is 271 based on a 5% margin of error, 90% confidence level, and response distribution of 50% [22]. The study was approved by the Bioethics Committee of Scientific and Medical Research, University of Jeddah, Saudi Arabia (UJ-REC-010).

### 2.2. Study Questionnaire

A self-administered online questionnaire was used to collect data through a web link shared over email and social media, such as Facebook, Twitter, and WhatsApp. Participants were randomly invited through the official channels of the University of Jeddah and King Abdulaziz University. Google Forms was used to construct the questionnaire, which included closed-ended questions written in Arabic; the questionnaire took less than 5–7 min to complete. In the online survey on the first page, the inclusion criteria of study sample were clearly stated. The inclusion criteria were adults above 18 years of age living in Jeddah city, and it was asked that any person outside of the inclusion criteria not to fill the questionnaire. Information explaining the study objectives and the target population was included on the first page of the questionnaire. The first page of the questionnaire also confirmed voluntary and confidential participation in the study. Signed online consent forms were obtained from all participants prior to data collection. The survey was anonymous, there was no collection of sensitive information, and the participants were able to leave at any point if they felt uncomfortable. The questionnaire was composed of three sections: sociodemographic characteristics of the participants (that is, sex, age, nationality, marital status, education level, occupation, and household monthly income), understanding of, and concern over climate change, and dietary choices.

### 2.3. Assessment of Understanding of and Concern over Climate Change

The second part of the survey included participant understanding and concern regarding climate change. The questionnaire was adapted from Korkala et al. [1] and Hope [23] with slight modifications. The question, “What do you think is meant by climate change?” was used to evaluate participants’ understanding of climate change. Five options were provided: (1) “an increase in sunspot activity and solar radiation,” (2) “a change in the axial tilt of the Earth,” (3) “an increase in population growth, energy consumption, and exploitation of nature,” (4) “an increase in the greenhouse gas concentration of the atmosphere due to human actions,” and (5) “a natural fluctuation of climate periods on Earth.” Based on the IPCC report [24], alternatives 3 and 4 indicated sufficient understanding resulting in a score of 1, whereas alternatives 1, 2, and 5 indicated a poor understanding of climate change resulting in a score of 0. Furthermore, two questions were asked to assess the degree of concern over climate change. The first question was, “How serious of a threat do you think climate change is to humankind?” The choices were (1) “a very serious threat,” (2) “quite a serious threat,” (3) “not a serious threat,” (4) “not a threat at all,” and (5) “I do not know.” Alternatives 1 and 2 indicated a high level of concern, whereas alternatives 3, 4, and 5 indicated low concern over climate change. The second question was, “What are the possible future effects of climate change in Saudi Arabia?” The choices included (1) “hotter temperatures,” (2) “more rain and floods,” (3) “colder temperatures,” (4) “rise in sea level,” (5) “loss in animals and plants,” (6) “no effect,” and (7) “I do not know.” Alternatives 1 to 5 indicated a high level of concern, while alternatives 6 and 7 indicated low concern over climate change. The answers that reflect high levels of concern were given a score of 1 and the sum of scores for the two questions was calculated for all participants.

### 2.4. Assessment of Dietary Choices

The third part of the questionnaire collected information regarding food consumption during the past 12 months via a short food frequency questionnaire (FFQ) with 14 items adapted from a previous study [1]. The food items were selected based on their climate-friendliness; the FFQ included both climate-friendly and non-climate-friendly food items. The climate-friendliness of food items has been defined in previous studies based on their GHGEs. Climate-friendly foods include fresh fruits [25], fresh vegetables/salad/root vegetables [25,26], potatoes (cooked or mashed) [25], vegetable oil [25], margarine [27], soy products (such as tofu) [28], and organic foods [29,30]. Non-climate-friendly food items include French fries [31], rice [25], beef/lamb [25,26], poultry [25], milk [25], low-fat cheese, other cheeses [25], and butter [27]. The calculation to determine climate-friendly food consumption involved summing the intake of fresh fruits, fresh vegetables, potatoes (cooked or mashed), vegetable oil, margarine, soy products (such as tofu), and organic food divided by seven. The calculation for non-climate-friendly food consumption involved summing the intake of French fries, rice, beef/lamb, poultry, milk, low-fat cheese, other cheeses, and butter divided by eight.

### 2.5. Statistical Analysis

All statistical analyses were performed using the statistical analysis software program SPSS version 27. Descriptive statistics, including percentage and frequency distributions, were calculated from the data. The scores for the understanding of, and concern about climate change and food intake are presented as the mean± standard deviation (SD). The score for understating and concerns were stratified to low and high; a high score for understating reflects participants with a score of one, and for concerns reflects participants with a score of two. A regression model was used to determine differences in the understanding and concern scores between participants according to their sociodemographic data adjusted for sex, age group, nationality, marital status, education, occupation, and income. Food scores were also compared with understanding of, and concern about climate change via a linear regression analysis adjusted for sex, age group, nationality, marital status, education, occupation, and income. Statistical significance was set at *p* ≤ 0.05.

## 3. Results

A summary of the participant sociodemographic characteristics is presented in Table 1. Nearly one-third of the study participants were between the ages of 18–29 years of age, while a quarter were between 30 and 39 years of age and a quarter were between 40 and 49 years of age. The majority of the study participants were females (66%), Saudis (95%), married (57%), and employed in the government sector (40%). Almost 61% of the study participants held a bachelor’s degree, and 32% had a postgraduate’s degree. A total of 36% of the study participants earned less than SAR 3000 monthly, and 21% earned SAR 7000–12,000 monthly.

Nearly 87% of study subjects acknowledged that they had heard about climate change, whereas a small percentage (13%) said they had never heard about it. Likewise, approximately half of the study participants (45%) stated that they understood the term climate change, and 39% said they understood it to some extent. In contrast, only a small percentage (16%) said they did not know what climate change meant. In addition, 77% of the participants believe that human activity is largely responsible for climate change, while 10% believe that human activity is not responsible for climate change and a further 13% are unsure whether human activity is responsible or not (Table S1). Overall, 45% of the study participants demonstrated a sufficient understanding of climate change and 55% exhibited a poor understanding. Approximately 56% of study participants displayed a high level of concern over climate change and 44% exhibited low concern.

Climate change understanding and concern scores stratified by sociodemographic characteristics are presented in Table 2. Regarding the climate change understanding score, there were significant differences between males and females (*p* < 0.0001), where males displayed a higher understanding score (0.71 ± 0.45) compared with females (0.31 ± 0.46). There were no significant differences in the climate change understanding score between participants in different age, nationality, marital status, education, occupation, and monthly income groups. Regarding the concern over climate change score, males demonstrated significantly greater concern over climate change scores (1.59 ± 0.70) compared with females (1.35 ± 0.69; *p* = 0.01). Additionally, participants with a postgraduate degree demonstrated the greatest concern about climate change scores compared with the other two educational groups (*p* = 0.006). There were statistically significant differences in concern over climate change scores between participants in different income and occupational groups (*p* = 0.03 and *p* = 0.01, respectively). Participants working in the public sector and with a monthly salary between SAR 7000 and 12,000 exhibited the highest level of concern over climate change scores. There were no significant differences in concern over the climate change score between participants in different age, nationality, and marital status groups.

Table 3 displays participants’ intake of selected food items according to their understanding of the climate change score. The participants were categorized as low (*n* = 154) and high (*n* = 126) according to their understanding of the climate change score. There was a significant positive association between understanding of climate change score and vegetable oil intake when comparing participants with high scores (3.26 ± 1.07) to those with low scores (3.00 ± 1.01; *p* = 0.009). Additionally, participants in the high score group consumed more vegetables (3.47 ± 0.98) compared with participants in the low score group (3.31 ± 0.96) after adjustment (*p* = 0.01). There were no significant differences between the intake of fruits, French fries, potatoes, rice, red meat, poultry, milk, low-fat cheese, other cheeses, butter, margarine, soy products, and organic food, and understanding of climate change score. 

Participants’ intake of selected food items according to their concern over climate change scores is presented in Table 4. Participants were categorized according to their concern over climate change scores into low (*n* = 123) and high (*n* = 157) groups. Higher scores for concerns about climate change were negatively associated with the intake of red meat (*p* = 0.0001), poultry (*p* = 0.003), margarine (*p* = 0.02), and soy products (*p* = 0.04). The intake of red meat, poultry, margarine, and soy products among participants in the high score group were lower (2.71 ± 0.95, 3.17 ± 0.88, 2.15 ± 1.04, and 1.82 ± 0.97, respectively) than that in the low score group (2.98 ± 0.74, 3.38 ± 0.65, 2.38 ± 1.25, and 2 ± 1.07, respectively). There were no significant differences between the intake of fruits, vegetables, French fries, potatoes, rice, milk, low-fat cheese, other cheeses, butter, vegetable oil, and organic food, and concern over climate change score. Overall, the participant intake of non-climate-friendly food was higher than that of climate-friendly food (2.93 ± 0.56 and 2.58 ± 0.60, respectively).

## 4. Discussion

Climate change is a major threat to public health. It has critically damaged various life support systems and cycles, endangering human lives. Dietary choices play a significant role in reducing climate change and alleviating its environmental impacts [5,14]. The current study investigated the levels of understanding and concern about climate change in Saudi Arabia and examined their associations with sociodemographic characteristics. It also examined the associations between the study participants’ levels of understanding and concern about climate change and dietary choices. The results revealed a poor understanding of climate change but high concern among the study participants. In addition, males and medium-high monthly income individuals were associated with higher understanding of, and concern over climate change scores. Similarly, higher education and government employees were associated with higher concern over climate change scores. Furthermore, subjects with a higher understanding of climate change scores exhibited a higher consumption of vegetables and vegetable oils. In addition, subjects who demonstrated higher concern over climate change scores exhibited lower red meat, poultry, margarine, and soy product consumption.

Although 87% of the study participants had heard about climate change and 45% admitted that they understood the term climate change, only 45% of study participants demonstrated a sufficient understanding of climate change. A poor understanding of climate change was reported in Bangladesh [32,33,34], whereas a greater understanding of climate change was reported in Finland [1]. In the current study, some participants expressed an understanding of human activities being responsible for the rise in GHGEs (39%); however, more (44%) believed that climate change is a natural fluctuation of climate periods on Earth, which reflects a poor understanding of climate change. In contrast, the study participants exhibited high levels of concern over climate change; nearly 62% of participants believed that climate change is a major threat to humans. A previous study reported that 36% of the Finnish population had high concerns and 47% demonstrated medium concerns about climate change [1]. Several studies have found high levels of concern over climate change [32,34], which is consistent with our findings.

This study demonstrated that males exhibited a good understanding and greater concern over climate change than females, even though the number of females participating in the study was higher than that of males (66 and 34%, respectively). Previous studies have demonstrated a greater female understanding of, and concern over climate change than males [1,23,33]. A previous study in Turkey determined that females were more concerned about climate change than males [33]. Similarly, a study in Finland illustrated that females exhibited a superior understanding and awareness of climate change compared with males [1]. However, a study in Guyana found no significant difference between both sexes in their understanding of climate change [23]. Scientific evidence has indicated sex differences regarding obtaining health knowledge and adopting healthy behaviors since females are more conscientious and more likely to seek reliable health information [35,36]. Our results can be explained by the fact that the males were more educated and older than the females in this study; 40% of males were postgraduate degree holders, whereas only 28% of females held a postgraduate degree. In addition, 39% of males in the study were 40–59 years of age, whereas 35% of females were 18–29 years of age. Thus, since the males in our sample were older and more educated than the females, they may be more knowledgeable about climate change topics and news.

Moreover, previous research indicated that the education level is a strong predictor of climate change knowledge and awareness [32,33], which aligns with our findings that participants with higher education levels (postgraduate degree) were highly concerned about climate change. A study from Bangladesh demonstrated that subjects with a sufficient level of education were more aware of climate change [32]. Other studies from the Philippines, Nepal, and China have reported similar results [37,38,39]. Therefore, increasing knowledge about climate change among students is a valuable strategy that can be used to enhance awareness of climate change. Furthermore, the results of this study indicate that a higher income is associated with a greater concern about climate change. One study from China illustrated that low-income individuals were less aware of climate change than those with a high income [39]. This is likely because individuals with higher income levels can afford higher levels of education.

The current study results demonstrated that a sufficient understanding of, and higher concern over climate change were associated with a greater intake of vegetables and vegetable oils and a lower intake of red meat, poultry, margarine, and soy products among the study subjects. These results corroborate with those of previous studies, demonstrating that concerns about the environment are associated with sustainable behaviors, such as increasing eco-friendly product consumption [1]. Specific food item consumption and production are a primary cause of GHGEs [40]. Previous research indicated that animal products, such as red meat, poultry, cheese, and butter are associated with high GHGEs, whereas fruits, vegetables, and margarine are associated with low GHGEs and can thus be considered climate-friendly food items [25,31,40]. A study in Finland illustrated that individuals who demonstrated sufficient knowledge about climate change exhibited a higher intake of fruits, vegetables, vegetable oils, organic foods, and soy products. The study also indicated that individuals with a high level of concern over climate change consumed less red meat and French fries; however, they consumed more fruits, vegetables, vegetable oils, organic foods, and soy products [1]. These findings are consistent with our results, except for soy product and organic food intake; the consumption of soy products and organic foods was low in our study. Nearly half of the study subjects (48%) reported that they never consumed soy products or consumed them less than once per month in the last 12 months. The consumption of organic foods was also low; 20% of participants admitted that they never bought organic food, and 40% stated that they consumed organic food less than once per month in the last 12 months. One possibility for this finding is the higher prices of these food items in Saudi Arabia, particularly organic food.

Food and livestock production is the primary cause of environmental harm, since it typically consists of excess water use and deforestation [4]. For example, the production of meat and dairy products is responsible for 15–24% of GHGEs worldwide, which explains why climate change scientists consider these food items non-climate friendly [4,35]. In the United Kingdom, one study indicated that reducing meat consumption decreased the production of GHGEs by 35%. In addition, eliminating food waste also decreased GHGEs by 12% [41]. Similarly, a study in the United States suggested that replacing meat with plant-based food effectively reduced GHGEs and improved human health [42]. Research demonstrated that vegan and vegetarian diets are highly associated with reduced GHGEs compared with a diet rich in meat (including fish) [43]. A study in Lebanon indicated that the western diet, which is rich in meat, dairy products, and processed foods, was highly associated with GHGEs. In contrast, the Mediterranean diet, which is rich in vegetables, fruits, and legumes, produced minor amounts of GHGEs [44]. Our results demonstrated that individuals with higher concern over climate change scores exhibited significantly lower red meat and poultry consumption. This implies that the effect of concern on individual behaviors, including environmental behaviors, may be higher than the effect of knowledge on behaviors that has been demonstrated in previous research [1,45]. However, our results also showed that individuals with a higher concern of climate change score had no increase in the intake of vegetables. Indeed, previous studies reported that the population of Saudi Arabia had a generally insufficient intake of fruits and vegetables [46,47], which may be the reason for this finding.

Climate-friendly foods can also improve human health and prevent chronic diseases; hence, people may increase their intake of these food items for health purposes instead of climate change mitigation. Numerous studies have demonstrated that reducing the consumption of red meat prevents coronary heart disease, type 2 diabetes, and several types of cancers, in addition to decreasing the production of GHGEs [48,49]. Chronic diseases, such as coronary heart disease, obesity, hypertension, high blood lipids, and type 2 diabetes, are highly prevalent in Saudi Arabia [50]. Saudi Arabia ranked number 84 in the Global Climate Risk Index (2018) since it has many environmental challenges that place the Kingdom at great risk. These challenges include air pollution, restricted freshwater sources, coastal flooding, high rates of energy consumption, and high rates of CO_2_ emissions per capita [51]. The Kingdom of Saudi Arabia has implemented numerous strategies for climate change mitigation, including green building projects and sustainable transportation, such as light rail transit in Riyadh, Jeddah, Makkah, Madinah, and Dammam, as well as the Riyadh metro [51]. The “Green Saudi initiative” was launched in 2021 as part of the national 2030 vision. The initiative aims to increase vegetation cover and reduce land degradation, air pollution, and CO_2_ emissions nationwide [52]. Regardless of the massive efforts of the Saudi government to mitigate climate change, the study indicated poor understanding and medium concern over climate change in the Kingdom. Therefore, enhancing the knowledge and concerns about climate change, increasing the intake of climate-friendly food choices, and reducing or replacing the intake of non-climate-friendly food among the population of Saudi Arabia can simultaneously benefit human and environmental health.

### 4.1. Limitations and Future Studies

The current study has several limitations. The FFQ required study participants to accurately remember their food intake in the last 12 months, introducing some recall bias. In addition, information such as the portion size of the food and the amount of food wasted was absent, which is considered a weakness of this study, since this information is highly related to reducing climate change. Further, the results cannot be generalized to all Saudis, since the study was only conducted in one city. Future studies should include representative samples from all regions of the country. In addition, using an online survey to recruit study participants may introduce self-selection bias and non-probability sampling; however, the online survey was a safe way to collect the data for this study due to the COVID-19 pandemic. Similarly, selection bias may be an issue in this study because participants who were interested in the topic “dietary choices as determinants of the understanding and concerns over climate change in Saudi Arabia” might have been more enthusiastic to complete the survey.

Moreover, this study did not assess the participant awareness of the relationship between food choices and climate change issues. Therefore, their consumption of specific food items may be related to managing health instead of climate change mitigation. A previous study in Saudi Arabia reported that females were unaware of the impact of their food choices on the environment [19]. Future studies should also explore the barriers to organic food consumption in Saudi Arabia and examine the Saudi attitudes and behaviors surrounding eco-friendly food consumption. Further research is needed to determine whether the decrease in meat consumption is related to the growing level of concern.

This study also exhibits several strengths. This is the first study to examine the associations between the understanding of and concern over climate change and dietary choices in Saudi Arabia to the best of our knowledge. The study included many food items (14 items) that were considered either climate-friendly or non-climate-friendly food items based on previous research. The findings of the current study offer insight into how individuals of Saudi Arabia understand and believe in climate change issues. It also provides a foundation for future studies to promote healthy dietary choices and mitigate climate change in the country.

### 4.2. Managerial Implications

We proposed the following recommendations based on our results. Public health representatives should increase awareness of the impact of climate change on public health. Universities and schools should also play a pivotal role in protecting the environment. Specific curricula should be dedicated to educating students about the importance of protecting the environment and how sustainable behaviors can mitigate the impact of their consumption on the environment. In particular, initiatives and campaigns that disseminate knowledge about individual dietary habits and their subsequent influence on the environment and public health should be increased. Further efforts should be made by the Ministry of Environment, Water, and Agriculture to increase Saudi population awareness of the benefits of sustainable consumption on the environment and to broaden their knowledge about their responsibility toward the environment. Businesses, as part of their social responsibility, should encourage green products, services, and operations to foster green consumption behaviors. These efforts can be supported by social marketing campaigns that foster and encourage the consumption of green products and services among Saudi consumers.

Expanding communication through social media, such as Twitter, Facebook, Instagram, and Snapchat, is the most common approach used by Saudis to disseminate information. Specific campaigns can be directed to Saudi females to increase their knowledge about the negative impact of climate change on the environment and health and encourage their engagement to protect the environment. Governments, businesses, and communities should work together to enhance awareness of the benefits of climate-friendly food choices for climate change mitigation and public health, to encourage sustainable behaviors.

## 5. Conclusions

Climate change is a global phenomenon that threatens human health, biodiversity, and the environment. The extent to which human practices and behaviors have an impact on the environment, and specifically climate change, has been documented in the literature; choosing what to eat is one of them. In particular, dietary choices have an enormous impact on the environment and are important strategies to combat climate change. Hence, the current study investigates climate change awareness and concern in Saudi Arabia, as well as the association between these factors, social demographics, and dietary choices. Data were collected for this cross-sectional research using an online questionnaire. We retrieved 280 completed forms; 45% of the study participants had sufficient understanding of climate change, and 56% were concerned about climate change. Participants who understand climate change and are concerned about it are more likely to be male, have a medium-high monthly income, have a high education level, and work for the government. The findings also revealed that participants with a sufficient understanding of climate change ate more vegetables and vegetable oils, while those with a high level of concern for climate change ate less red meat, poultry, margarine, and soy products. Furthermore, the intake of non-climate-friendly food was typically higher than the intake of climate-friendly food among the study participants. These findings are critical for encouraging people to adopt healthy behaviors and consume climate-friendly food to minimize the risk of climate change and improve public health. The current study contributes to the body of literature about climate change by investigating the relationship between food intake and its subsequent impact on the environment. As far as we are aware, this is the first study of its kind to measure Saudi citizens’ awareness of the effects of climate change and its impact on their food choices. The findings of this study shed light on how citizens in Saudi Arabia perceive and believe in climate change. As a result, we have a better understanding of Saudi Arabians’ perspective about climate change. It also lays the groundwork for future studies aimed at promoting healthy eating habits and reducing climate change in the country.

## Figures and Tables

**Table 1:** Participants’ sociodemographic characteristics.

Characterization	Frequency (%)
Sex	
Male	96 (34.3)
Female	184 (65.7)
Age group	
18–29	93 (33.2)
30–39	74 (26.4)
40–49	74 (26.4)
50–59	33 (11.8)
≥60	6 (2.1)
Nationality	
Saudi	265 (94.6)
Others	15 (5.4)
Marital status	
Single	99 (35.4)
Married	160 (57.1)
Divorced/widow	21 (7.5)
Education	
High school or lower	20 (7.1)
Bachelor’s degree	170 (60.7)
Postgraduate	90 (32.1)
Occupation	
Student	52 (18.6)
Government employ	109 (39.9)
Private employ	40 (14.3)
Free work	10 (3.6)
Retired	12 (4.3)
Unemployed	57 (20.4)
Monthly income (SAR)	
<3000	101 (36.1)
3000–7000	38 (13.6)
7000–12,000	58 (20.7)
12,000–20,000	33 (11.8)
>20,000	50 (17.9)

**Table 2:** Participants’ understanding of climate change and concern about climate change scores stratified based on sociodemographic data.

Characterization	Understanding Score	*p* Value	Concern Score	*p* Value
**Sex ^1^**				
Male	0.71 ± 0.45	**<0.0001**	1.59 ± 0.70	**0.01**
Female	0.31 ± 0.46		1.35 ± 0.69	
**Age group ^2^**				
18–29	0.41 ± 0.49	0.34	1.38 ± 0.65	0.07
30–39	0.45 ± 0.50		1.59 ± 0.61	
40–49	0.41 ± 0.49		1.36 ± 0.75	
50–59	0.63 ± 0.48		1.36 ± 0.85	
≥60	0.33 ± 0.51		1.50 ± 0.83	
**Nationality ^3^**				
Saudi	0.44 ± 0.49	0.66	1.42 ± 0.70	0.18
Others	0.53 ± 0.51		1.60 ± 0.73	
**Marital status** **^4^**				
Single	0.43 ± 0.49	0.98	1.52 ± 0.57	0.27
Married	0.46 ± 0.50		1.38 ± 0.76	
Divorced/widowed	0.38 ± 0.49		1.42 ± 0.74	
**Education ^5^**				
High school or lower	0.45 ± 0.51	0.20	1.25 ± 0.71	**0.006**
Bachelor’s degree	0.40 ± 0.49		1.36 ± 0.73	
Postgraduate	0.54 ± 0.50		1.61 ± 0.61	
**Occupation ^6^**				
Student	0.44 ± 0.50	0.64	1.57 ± 0.53	**0.01**
Government employ	0.49 ± 0.50		1.59 ± 0.61	
Private employ	0.42 ± 0.50		1.27 ± 0.71	
Free work	0.60 ± 0.51		1.40 ± 0.84	
Retired	0.33 ± 0.49		1.00 ± 0.95	
Unemployed	0.38 ± 0.49		1.21 ± 0.81	
**Monthly income (SAR) ^7^**				
<3000	0.34 ± 0.47	0.24	1.34 ± 0.71	**0.03**
3000–7000	0.39 ± 0.49		1.28 ± 0.69	
7000–12,000	0.55 ± 0.50		1.68 ± 0.53	
12,000–20,000	0.63 ± 0.48		1.48 ± 0.79	
>20,000	0.46 ± 0.50		1.40 ± 0.75	

^1^ *p* Value was analyzed using regression model adjusted for age, nationality, marital status, education, occupation, and income; ^2^ *p* value was analyzed using regression model adjusted for sex, nationality, marital status, education, occupation, and income; ^3^ *p* value was analyzed using regression model adjusted for sex, age, marital status, education, occupation, and income; ^4^ *p* value was analyzed using regression model adjusted for sex, age, nationality, education, occupation, and income; ^5^ *p* value was analyzed using regression model adjusted for sex, age, nationality, marital status, occupation, and income; ^6^ *p* value was analyzed using regression model adjusted for sex, age, nationality, marital status, education, and income; ^7^ *p* value was analyzed using regression model adjusted for sex, age, nationality, marital status, education, and occupation. Significant values are bolded.

**Table 3:** Participants’ intake of selected food items according to their understanding of climate change score.

Food Items	Low Score (*n* = 154)	High Score (*n* = 126)	Adjusted *p* Value
Fruits	2.88 ± 1.01	3.03 ± 1.05	0.18
Vegetables	3.31 ± 0.96	3.47 ± 0.98	**0.01**
French fries	2.41 ± 0.84	2.52 ± 1.00	0.80
Potatoes	2.03 ± 0.92	2.14 ± 0.99	0.29
Rice	3.14 ± 0.86	3.31 ±0.87	0.26
Red meat	2.87 ± 0.82	2.77 ± 0.94	0.05
Poultry	3.27 ± 0.75	3.26 ± 0.85	0.29
Milk	3.31 ± 0.95	3.44 ± 1.00	0.33
Low-fat cheese	2.80 ± 1.08	2.97 ± 1.04	0.11
Other cheeses	2.98 ± 1.03	3.04 ± 1.09	0.40
Butter	2.33 ± 0.89	2.49 ± 1.04	0.33
Vegetable oil	3.00 ± 1.01	3.26 ± 1.07	**0.009**
Margarine	2.27 ± 1.18	2.23 ± 1.09	0.64
Soy products	1.95 ± 1.03	1.83 ± 1.00	0.25
Organic food	2.27 ± 1.01	2.50 ± 1.07	0.64

*p* Value was analyzed using univariate regression adjusted for sex, age group, nationality, marital status, education, occupation, and income. Probability < 0.05 is considered significant. Significant values are bolded.

**Table 4:** Participant intake of selected food items according to their concern over climate change score.

Food Item	Low Score (*n* = 123)	High Score (*n* = 157)	Adjusted *p* Value
Fruits	2.91 ± 0.97	2.98 ± 1.08	0.86
Vegetables	3.37 ± 0.91	3.39 ± 1.02	0.75
French fries	2.45 ± 0.83	2.47 ± 0.99	0.32
Potatoes	2.17 ± 0.96	2.01 ± 0.95	0.12
Rice	3.24 ± 0.83	3.21 ± 0.91	0.26
Red meat	2.98 ± 0.74	2.71 ± 0.95	**0.0001**
Poultry	3.38 ± 0.65	3.17 ± 0.88	**0.003**
Milk	3.39 ± 0.81	3.35 ± 1.08	0.41
Low-fat cheese	2.79 ± 1.05	2.94 ± 1.07	0.25
Other cheeses	2.99 ± 0.99	3.02 ± 1.11	0.77
Butter	2.39 ± 0.92	2.41 ± 0.99	0.73
Vegetable oil	3.14 ± 0.96	3.09 ± 1.11	0.68
Margarine	2.38 ± 1.25	2.15 ± 1.04	**0.02**
Soy products	2 ± 1.07	1.82 ± 0.97	**0.04**
Organic food	2.31 ± 1.07	2.43 ± 1.02	0.99

*p* Value was analyzed using univariate regression adjusted for sex, age group, nationality, marital status, education, occupation, and income. Probability < 0.05 is considered significant. Significant values are bolded.

## Data Availability

The datasets generated and/or analyzed during the current study are not publicly available because the authors require them for further publication. They are available from the corresponding author upon reasonable request. Signed online consent forms were obtained from all participants prior to data collection.

## Supplementary Materials

The following are available online at https://www.mdpi.com/article/10.3390/ijerph182010605/s1, Table S1: Participant responses to climate change questions.
