# Supplementary material for: Sociodemographic Characteristics and Dietary Choices as Determinants of Climate Change Understanding and Concern in Saudi Arabia
Source: Int J Environ Res Public Health. 2021 Oct 10;18(20):10605. doi: 10.3390/ijerph182010605 (PMC8535203; doi:10.3390/ijerph182010605)
Supplement: Supplementary file 1 [file ijerph-18-10605-s001.zip › ijerph-1366261-supplementary.pdf]

Table S1. Participant responses to climate change questions

Questions	Frequency (%)
**Have you heard the term "climate change?"**	
Yes	243 (86.8)
No	37 (13.2)
**Do you understand what "climate change" means?**	
Yes	126 (45)
No	26 (9.3)
I do not know	19 (6.8)
To some extent	109 (39)
**What do you think is meant by "climate change?"**	
An increase in sunspot activity and solar radiation	12 (4.3)
A change in the axial tilt of the Earth	15 (5.4)
An increase in population growth, energy consumption, and exploitation of nature	20 (7.1)
An increase in the greenhouse gas concentration of the atmosphere due to human actions	110 (39.3)
A natural fluctuation of climate periods on Earth	123 (43.9)
**How serious of a threat do you think "climate change" is to humankind?**	
A very serious threat	116 (41.4)
Quite a serious threat	57 (20.4)
Not a serious threat	33 (11.8)
Not a threat at all	21 (7.5)
I do not know	53 (18.9)
**Do you think human activity is largely responsible for "climate change?"**	
Yes	216 (77)
No	28 (10)
I do not know	36 (13)
**What are the possible future effects of "climate change" in Saudi Arabia?**	
Hotter temperatures	132 (47.1)
More rain and floods	17 (6.1)
Colder temperatures	29 (10.4)
Rise in sea level	13 (4.6)
Loss of animals and plants	38 (13.6)
No effect	2 (0.7)
I do not know	49 (17.5)
